# Draft Genome Sequence of the Putative Endophytic Bacterium Pantoea agglomerans R6, Associated with Lactuca serriola from South Africa

**DOI:** 10.1128/MRA.00023-21

**Published:** 2021-03-11

**Authors:** Brandon Istain, Prudent S. Mokgokong, Ruomou Wu, Junaid Mia, Arun Gokul, David Mendoza-Cozatl, Morné Du Plessis, Marshall Keyster

**Affiliations:** a Environmental Biotechnology Laboratory, Department of Biotechnology, University of the Western Cape, Bellville, South Africa; b DST-NRF Centre of Excellence in Food Security, University of the Western Cape, Bellville, South Africa; c Zoological Research, Foundational Research and Services, South African National Biodiversity Institute, Pretoria, South Africa; d Department of Biotechnology, University of the Western Cape, Cape Town, South Africa; e Division of Plant Sciences, C.S. Bond Life Sciences Center, University of Missouri, Columbia, Missouri, USA; University of Maryland School of Medicine

## Abstract

Here, we present the draft genome sequence (∼4.7 Mb) of the endopyhtic bacterium Pantoea agglomerans strain R6, which was isolated from surface-sterilized roots of Lactuca serriola (prickly lettuce).

## ANNOUNCEMENT

Pantoea agglomerans is a Gram-negative aerobic bacillus that belongs to the family *Erwiniaceae* and can be isolated from a wide variety of environments (1, 2). In plants, various strains were found to be either pathogens or commensals (3) and possess many beneficial agricultural traits (4).

In this study, *P. agglomerans* strain R6 was isolated from surface-sterilized roots of Lactuca serriola. The *L. serriola* root tissues were collected from the University of the Western Cape in Bellville, South Africa (33°56′06.0″S, 18°37′41.0″E), in April 2019. Clean root samples were sterilized with 70% ethanol for 3 min, 2.5% sodium perchlorate for 5 min, and 70% ethanol for 1 min and washed 5 times with autoclaved distilled water. The sterilized roots were crushed using a sterile mortar and pestle in a 0.9% saline (NaCl) solution, followed by incubation at 22°C for 5 h. The solution was diluted and spread plated (50 μl) onto R-2A agar (Sigma; catalog number 17209) and incubated at 22°C for 5 days. Following incubation, single colonies were picked and subcultured until a pure culture was obtained. For genomic DNA (gDNA) extraction, a single pure colony was cultured in nutrient broth (5 ml) at 22°C. The total genomic DNA was extracted with the GenElute bacterial genomic DNA kit (Sigma; catalog number NA2110). The purity and concentration of the DNA were measured using a NanoDrop 2000 spectrophotometer (Thermo Scientific).

Genomic DNA (100 ng) was fragmented using the Covaris ultrasonicator. The DNA was end repaired using the Ion Xpress Plus fragment library kit (Thermo Fisher Scientific), and the 2% agarose E-gel system was used to size select fragments larger than 300 bp. The gDNA size was confirmed using the high-sensitivity D1000 DNA ScreenTape kit on the Agilent TapeStation 4200 (Agilent Technologies). Libraries were constructed using the Ion Xpress kit (Thermo Fisher Scientific) and quantified using the QuantStudio 12K system using the Ion library TaqMan quantitation kit (Thermo Fisher Scientific). The gDNA libraries were diluted to 100 pM and subjected to emulsion PCR using the Ion 520 and Ion 530 Kit-Chef (Thermo Fisher Scientific). The final enriched libraries were loaded onto the Ion 530 chip kit and subjected to sequencing on the Ion Torrent 5S GeneStudio system.

This generated 2,219,537 single-end reads with a mean read length of 280 bp. Default parameters were used for all software, unless otherwise stated. FastQC v0.10.1 (5) was used for read quality assessment, where an average base quality of 30 was observed across all bases. The raw reads were trimmed for removal of 20 bp at the 5′ end along with selection for reads between 220 bp and 300 bp, using Trimmomatic v0.36 (6). In addition, reads with low GC content (<45%) were also removed from further analysis to prevent the generation of spurious results.

Assembly was performed using SPAdes v3.13.0 (7). The final draft assembly contained 366 contigs with selection for only contigs greater than 1,000 bp. The assembly covered a total of 439, 942, and 830 bp with an *N*_50_ value of 25,969 and GC content of 55.61%. The depth of sequencing for the assembly was calculated at approximately 100× coverage. The 16S rRNA gene of R6 was used to perform a BLAST search against the NCBI nonredundant nucleotide database, which returned Pantoea agglomerans as the closest identity.

The genome sequence and associated annotation contribute to the expanding database of *Pantoea* species that have been identified to date (8). The output serves as the foundational data to further explore the utility of the features of the sequenced genome, particularly in the context of its endophytic relationship.

### Data availability.

The assembled genome sequence of Pantoea agglomerans R6 is available at GenBank under the accession number JACRVA000000000 (version 1) with BioProject number PRJNA647595, BioSample number SAMN15595211, and SRA accession number SRR12904959.
